# Comparison of manual and artificial intelligence based quantification of myocardial strain by feature tracking—a cardiovascular MR study in health and disease

**DOI:** 10.1007/s00330-023-10127-y

**Published:** 2023-08-18

**Authors:** Jan Gröschel, Johanna Kuhnt, Darian Viezzer, Thomas Hadler, Sophie Hormes, Phillip Barckow, Jeanette Schulz-Menger, Edyta Blaszczyk

**Affiliations:** 1 Charité – Universitätsmedizin Berlin, corporate member of Freie Universität Berlin and Humboldt-Universität Zu Berlin, Berlin, Germany; 2 Working Group On Cardiovascular Magnetic Resonance, Experimental and Clinical Research Center, a joint cooperation between the Charité Medical Faculty and the Max-Delbrück Center for Molecular Medicine and HELIOS Hospital Berlin-Buch, Department of Cardiology and Nephrology, Berlin, Germany; 3 DZHK (German Centre for Cardiovascular Research), Partner Site Berlin, Berlin, Germany; 4 Circle Cardiovascular Imaging Inc, Calgary, AB Canada

**Keywords:** Feature tracking, Strain, Cardiovascular magnetic resonance, Artificial intelligence: reproducibility, Validation

## Abstract

**Objectives:**

The analysis of myocardial deformation using feature tracking in cardiovascular MR allows for the assessment of global and segmental strain values. The aim of this study was to compare strain values derived from artificial intelligence (AI)–based contours with manually derived strain values in healthy volunteers and patients with cardiac pathologies.

**Materials and methods:**

A cohort of 136 subjects (60 healthy volunteers and 76 patients; of those including 46 cases with left ventricular hypertrophy (LVH) of varying etiology and 30 cases with chronic myocardial infarction) was analyzed. Comparisons were based on quantitative strain analysis and on a geometric level by the Dice similarity coefficient (DSC) of the segmentations. Strain quantification was performed in 3 long-axis slices and short-axis (SAX) stack with epi- and endocardial contours in end-diastole. AI contours were checked for plausibility and potential errors in the tracking algorithm.

**Results:**

AI-derived strain values overestimated radial strain (+ 1.8 ± 1.7% (mean difference ± standard deviation); *p* = 0.03) and underestimated circumferential (− 0.8 ± 0.8%; *p* = 0.02) and longitudinal strain (− 0.1 ± 0.8%; *p* = 0.54). Pairwise group comparisons revealed no significant differences for global strain. The DSC showed good agreement for healthy volunteers (85.3 ± 10.3% for SAX) and patients (80.8 ± 9.6% for SAX). In 27 cases (27/76; 35.5%), a tracking error was found, predominantly (24/27; 88.9%) in the LVH group and 22 of those (22/27; 81.5%) at the insertion of the papillary muscle in lateral segments.

**Conclusions:**

Strain analysis based on AI-segmented images shows good results in healthy volunteers and in most of the patient groups. Hypertrophied ventricles remain a challenge for contouring and feature tracking.

**Clinical relevance statement:**

AI-based segmentations can help to streamline and standardize strain analysis by feature tracking.

**Key Points:**

• *Assessment of strain in cardiovascular magnetic resonance by feature tracking can generate global and segmental strain values.*

• *Commercially available artificial intelligence algorithms provide segmentation for strain analysis comparable to manual segmentation.*

• *Hypertrophied ventricles are challenging in regards of strain analysis by feature tracking.*

**Supplementary Information:**

The online version contains supplementary material available at 10.1007/s00330-023-10127-y.

## Introduction

Myocardial strain allows for a quantitative measurement of myocardial deformation. Analysis of strain using cardiovascular magnetic resonance (CMR) can be obtained either by tissue tagging or by direct feature tracking (FT) on standard cine images. While CMR tagging has been validated and has further advanced into various different techniques (e.g., (fast) Strain Encoding Magnetic Resonance imaging (SENC) or displacement encoding with stimulated echoes (DENSE)) [1–4], it still has the drawback of requiring special sequences and scan time. In contrast to this, FT is a promising tool as it allows for the assessment of segmental and global strains in longitudinal, circumferential, and radial directions (LS, CS, RS, GLS, GCS, and GRS, respectively) from standard cine images which are usually acquired during clinical routine [5]. Left ventricular (LV) strain analysis applying CMR has been implied in a wide array of clinical diseases ranging from chemotherapy-induced cardiotoxicity [6, 7] to ischemic heart disease [8–10] and even non-ischemic heart diseases like hypertrophic cardiomyopathies (HCMs) [11, 12] or cases of acute myocarditis [13, 14]. Despite its wide utility and power to detect myocardial changes even in states with preserved function, FT still lacks standardization and consensus about the methodological process. In a previous study, different factors, like post-processing software used, slice selection, and 2D or 3D analysis, which all have the potential to influence strain values, were analyzed [15]. Additionally, one must take the time-consuming manual contouring process as well as the reader’s level of expertise and training into consideration, which also impact strain evaluation [16]. One potential approach to reduce influence of manually derived contours is the use of artificial intelligence (AI)–derived contours. AI-based segmentation and strain evaluation have been previously applied and validated in a large cohort with commercially available software [17] as well as commercially unavailable software [18]. These advances may further streamline strain assessment and help to reach a consensus about a standardized approach to feature tracking in clinical routine and “big data” studies.

Our study aimed at evaluating and comparing manual and AI-based approaches regarding quantitative strain metrics used in clinical routine as well as on a contour level for strain assessment by FT in healthy volunteers and patients with different cardiac diseases in order to identify strengths and weaknesses of these methods.

## Materials and methods

The ethics review board approved all studies and all participants gave written informed consent.

### Study population

In the healthy volunteers cohort, 67 subjects, retrospectively recruited in a previous study [15], were included. For the final analysis, 7 volunteers had to be excluded due to lack of a short-axis (SAX) stack covering the entire ventricle and one due to significant respiratory artifacts, which ultimately resulted to a healthy cohort consisting of 60 subjects. For the clinical validation, a cohort of 76 patients, chosen from previous studies [19–21], with cases including left ventricular hypertrophy (LVH) (*n* = 46 consisting of 8 patients with arterial hypertension (AHT)), 24 with aortic stenosis (AS), 14 with HCM, and chronic myocardial infarction (CMI) (*N* = 30 consisting of 10 patients with preserved left ventricular ejection fraction (LVEF), no wall motion abnormalities (WMA) and focal fibrosis (CMI-F), 10 with reduced LVEF, regional WMA, focal fibrosis (CMI-WF), and 10 with reduced LVEF with dilated LVs, global WMA, and focal fibrosis (CMI-EWF)), was constructed.

### Imaging protocol

CMR was performed either at a 1.5-T scanner (MAGNETOM Avanto^−FIT^, Siemens Healthineers) or a 3-T scanner (MAGNETOM Verio, Siemens Healthineers). Steady-state free precession-based cine images were acquired for 3 long-axis (LAX) views including a 2 chamber view (cv), a 3 cv, and a 4 cv as well as one SAX stack covering the entire left ventricle. Sequence parameters for the SAX stack at the 1.5-T scanner were as follows: time of repetition 2.8–3.31 ms, slice thickness 7 mm with no gap, flip angle 80°, echo time 1.2–1.44 ms, field of view 340–380 × 276– 308, 75 mm^2^, matrix 192 × 156, voxel size 1.4–2.0 × 1.4–2.0, 30 cardiac phases; and for the 3-T scanner: time of repetition 3.1 ms, slice thickness 6 mm with no gap, flip angle 45°, echo time 1.3 ms, field of view 340 × 276 mm^2^, matrix 192 × 156, voxel size 1.4 × 1.4, 30 cardiac phases.

### Manual segmentations

Manual segmentation was performed with dedicated software (circle CVI^42^ version 5.14.7, Circle Cardiovascular Imaging Inc.). Manual endo- and epicardial contours were drawn in end-diastole (ED), determined by the phase with the largest LV volume in SAX as well as in the 2 cv, 3 cv, and 4 cv. We were particularly attentive during segmentation to avoid contouring phases with the left ventricular outflow tract (LVOT) still visible in diastole and/or systole. Papillary muscles were not separately contoured as recently published [15]. Reference points for the delineation of segments were manually placed at the subepicardial border at the anterior intersection of the left and right ventricle.

### AI-generated segmentations

Similarly to the manual strain assessment, AI contours were derived in ED. The AI segmented slices with visible LVOT using open LV endo- and epicardial contours, which were disregarded for strain analysis. Reference points were automatically set by the AI; however, each point was manually validated to obtain comparable segmental values. The AI segmentation algorithms employed in the Circle CVI^42^ software are comprised of different deep convolutional neural network models trained to perform SAX and LAX CMR image segmentation. A similar model architecture as that of the standard U-Net is adopted for this purpose, along with various data augmentation techniques to enhance the generalizability of the trained model. The model was trained on the UK Biobank data as well as datasets that include patient data with pathological conditions including tetralogy, cardiomyopathy, and hypertension [22]. These models operate solely on image pixel data and image header information such as image dimensions and pixel spacing.

### Strain assessment

After segmentation, a FT algorithm provided strain values. The algorithm uses myocardial points and tracks them along the cardiac cycle [23, 24]. On a quantitative level, the manual and the AI approach were compared for strain assessment in CS and RS retrieved from SAX and LS retrieved from LAX views. All strain values were derived for global as well as segmental values according to the 17-segment model of the American heart association (AHA) for CMR without the apical segment [25]. Correct FT was assessed by either mesh analysis or by tracking the myocardial points through the phases. Improper tracking was defined as mesh overlay or myocardial points not following the extent of the contours [15, 26]. To enable comparability between the segmentations regarding the strain analysis, we verified that the AI algorithm chose the proper ED phase.

### Statistical analysis

All continuous variables are presented by mean and standard deviation (SD). Normal distribution was visualized by QQ plots. A mixed model was used to assess measurement differences segmentally and globally between the modalities for healthy volunteers and patients combined. In the mixed model, a global test was applied to test for any differences. In the case of a significant global test, pairwise comparisons were performed. Additionally, we tested whether a difference found between the AI and manual segmentations was homogenous over all groups or whether a certain group showed major deviations.

Additionally, both segmentation approaches were compared using the “Lazy Luna” (LL) tool which allows to assess the similarity those of an experienced reader and an AI on the contour level, via reproducibility validation metrics [27]. We chose the Dice similarity coefficient (DSC) and the Hausdorff distance (HD) to compare the consensus of the manual contours and the AI approach. DSC scores were calculated based on myocardial class, which was derived from the intersection of the endo- and epicardial contours placed manually or by the AI. High DSC numbers signifying a substantial overlap of the segmented areas and low numbers indicate incongruences. Vice versa holds true for the HD metric. In order to compare the proper placement of the insertion point, the LL tool additionally compared the manual- and AI-placed insertion point based on an angular difference to the left ventricular centroid. As some SAX acquisitions were acquired after contrast media application (post-CM), GRS and GCS as well as DSC and HD metrics were compared with acquisitions pre-contrast media application (pre-CM). Statistical analysis was performed using dedicated software (SPSS version 26, International Business Machines and SAS version 9.4, SAS Institute Inc.). The segmentation comparison tool “Lazy Luna” and the bulls-eye plots were created in Python (Version3.8, Python Software Foundation) [27].

## Results

### Study population

**Fig. 1:**
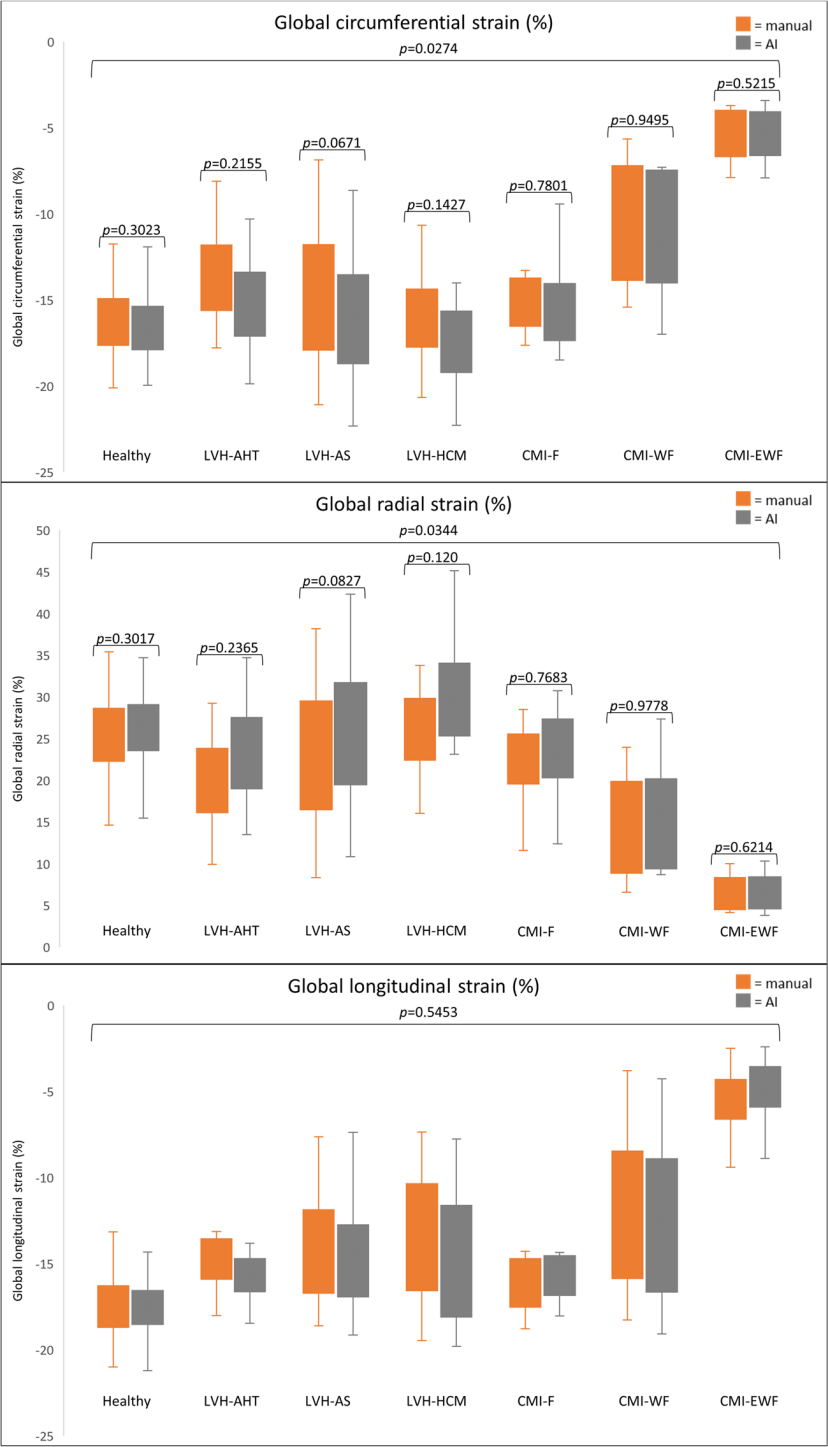
Global strain values for the healthy and the disease cohorts for circumferential, radial, and longitudinal strains in %. Overall, AI-derived strain values showed a trend towards overestimation of RS values and underestimation of CS and LS values. Global significant differences were found for circumferential and radial global strain values, with pairwise comparison revealing no significant differences. LVH, left ventricular hypertrophy; AHT, arterial hypertension; AS, aortic stenosis; HCM, hypertrophic cardiomyopathy; CMI, chronic myocardial infarction; F, cases with focal fibrosis, no wall motion abnormalities, and preserved left ventricular function; WF, cases with focal fibrosis, wall motion abnormalities, and reduced left ventricular function; EWF, cases with fibrosis, global wall motion abnormalities, and reduced left ventricular function and dilated left ventricles

In the healthy cohort, 67 subjects were re-analyzed and in the clinical cohort 76 patients. Details concerning the healthy volunteer cohort and the patients are given in Table 1. Overall, AI-derived strain values showed a trend towards overestimation of RS values ((mean difference % (± SD)) + 1.8 (± 1.7)) and an underestimation of CS (− 0.8 (± 0.8)) and LS values (− 0.1 (± 0.8)) (Fig. 1). Regarding the entire studied cohort, including the healthy probands and the ones with pathologies, global testing revealed significant differences for GCS (*p* = 0.03), GRS (*p* = 0.03), and RS AHA segments 5 (*p* = 0.045), 10 (*p* = 0.04), 11 (*p* = 0.002), and 12 (*p* = 0.03). Pairwise testing revealed no significant differences if manual and AI approaches were compared for the specific cohort for GCS, GRS, and RS AHA segment 12. Statistically significant results were found between AI and manual strain values for the subgroups AS and HCM for RS AHA segment 5 (*p* = 0.01 and *p* = 0.03, respectively), 10 (*p* = 0.01 and *p* = 0.02, respectively), and 11 (*p* = 0.02 and *p* = 0.01, respectively) (Supplementary material 1). Additionally, we found no significant interaction between the methods and the examined subgroups except for AHA segment 10 for CS (*p* = 0.02) as well as RS (*p* = 0.02) (Supplementary Material 2). Overall, in 83/136 cases (61%), SAX images were acquired post-CM. For the subgroups, the following percentages of SAX were acquired after post-CM: healthy cohort 37/60 (61.7%), CMI-F 10/10 (100%), CMI-WF 10/10 (100%), CMI-EWF 10/10 (100%), AHT 7/8 (87.5%), AS 8/24 (33.3%), HCM 1/12 (8.3%).

**Table 1:** Patient characteristics

Parameter*		Chronic myocardial infarction (*N* = 30)			Left ventricular hypertrophy (*N* = 46)		
Subgroup	Healthy volunteers	Focal fibrosis, no WMA and preserved LVEF	Focal fibrosis, regional WMA and reduced LVEF	Focal fibrosis, global WMA and dilated LV with reduced LVEF	Arterial hypertension	Aortic stenosis	Hypertrophic cardiomyopathy
*N* =	60	10	10	10	8	24	14
Gender (F/M)	26/34	4/6	1/9	0/10	4/4	7/17	3/11
Scanner (1.5 T/3 T)	36/24	10/0	10/0	10/0	8/0	14/10	12/2
Age (years)	44.1 ± 16.4	71.2 ± 8.5	68.6 ± 11.4	57.9 ± 12.3	64.0 ± 12.7	77.4 ± 7.5	48.4 ± 7.9
Height (cm)	173.8 ± 8.4	172.0 ± 9.2	174.6 ± 6.4	180.6 ± 7.5	172.5 ± 10.3	169.0 ± 8.1	178.2 ± 5.9
Weight (kg)	74.1 ± 12.4	76.5 ± 15.7	84.3 ± 9.6	92.9 ± 21.7	85.6 ± 14.6	86.1 ± 13.0	89.4 ± 7.0
BMI (kg/m^2^)	24.5 ± 3.8	25.6 ± 3.6	27.7 ± 3.3	28.3 ± 5.5	29.0 ± 6.0	29.3 ± 4.8	28.1 ± 1.6
BSA (m^2^)	1.9 ± 0.2	1.9 ± 0.2	2.0 ± 0.1	2.1 ± 0.3	2.0 ± 0.2	2.0 ± 0.2	2.1 ± 0.1
LVEDV (mL)	144.9 ± 30.8	163.0 ± 39.8	175.5 ± 59.1	303.3 ± 82.0	171.8 ± 55.1	150.1 ± 36,9	163.4 ± 32.8
LVESV (mL)	60.4 ± 17.5	77.5 ± 24.5	100.1 ± 53.1	236.6 ± 73.1	75.5 ± 27.3	63.9 ± 18.3	62.7 ± 13.6
LVSV (mL)	84.5 ± 17.2	85.4 ± 21.9	75.3 ± 13.5	66.7 ± 19.6	96.3 ± 29.1	86.2 ± 25.3	100.8 ± 25.4
LVEF (%)	58. 7 ± 6.1	52.9 ± 7.6	45.4 ± 13.5	22.6 ± 5.3	56.4 ± 4.0	57.3 ± 8.1	61.2 ± 6.4
LVM (g)	88.6 ± 20.1	114.5 ± 24.8	124.7 ± 20.3	162.9 ± 33.5	154.5 ± 58.2	136.7 ± 40.2	153.2 ± 52.8

*WMA* wall motion abnormalities, *LV* left ventricle, *LVEF* left ventricular ejection fraction, *BMI* body mass index, *BSA* body surface area, *LVEDV* left ventricular end-diastolic volume, *LVESV* left ventricular end-systolic volume, *LVSV* left ventricular stroke volume, *LVM* left ventricular mass; *data represented as frequencies for categorical variables and as mean ± standard deviation for continuous variables

### Strain analysis—healthy cohort

**Fig. 2:**
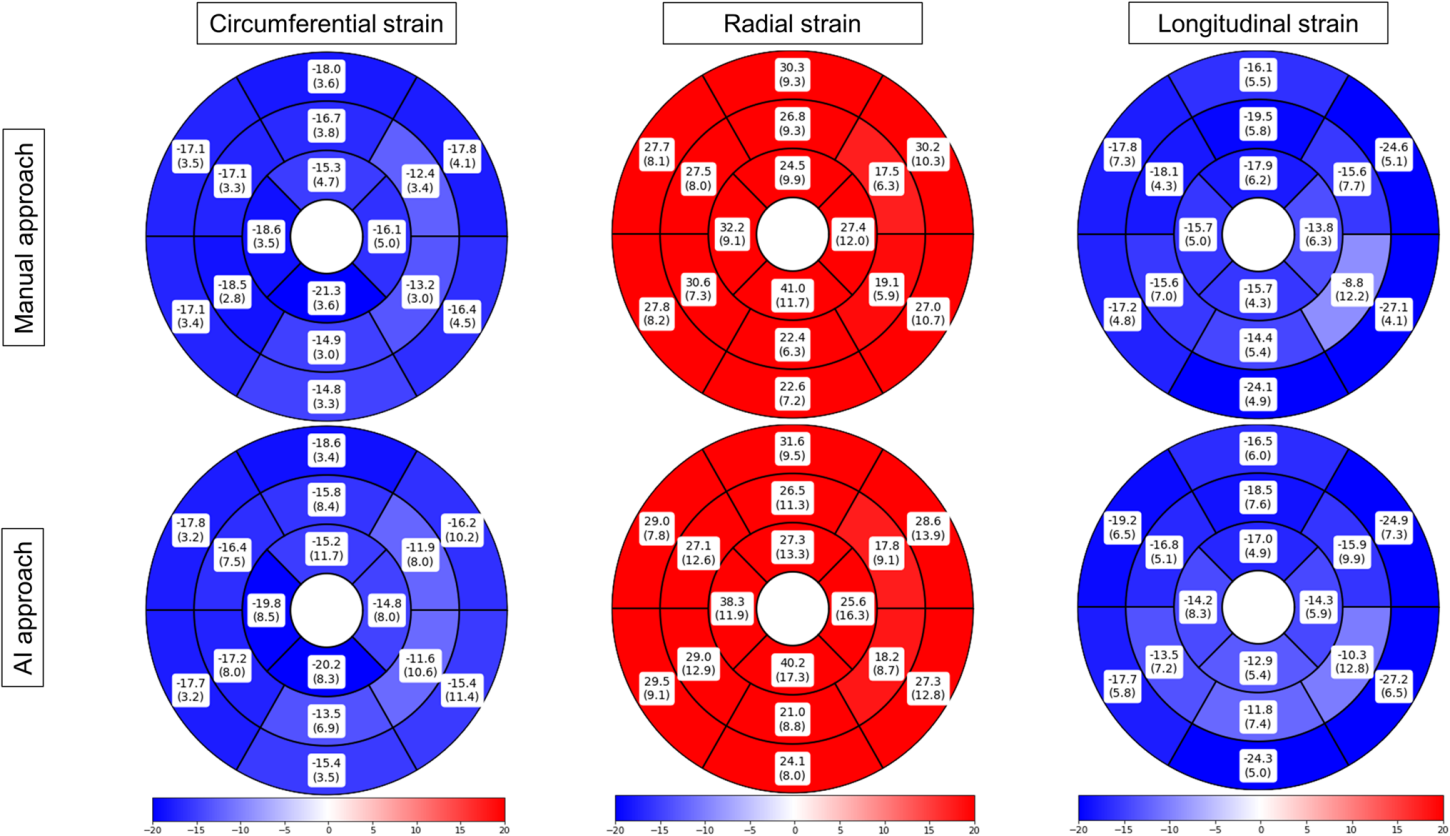
Segmental strain values for the healthy cohort for circumferential, radial and longitudinal strains in %. Segmental strain values, according to the American heart association model, are presented from left to right for circumferential, radial, and longitudinal strains in %. Pairwise comparisons showed no statistically significant differences between the manual (top) and the AI-based (bottom) segmentations

**Fig. 3:**
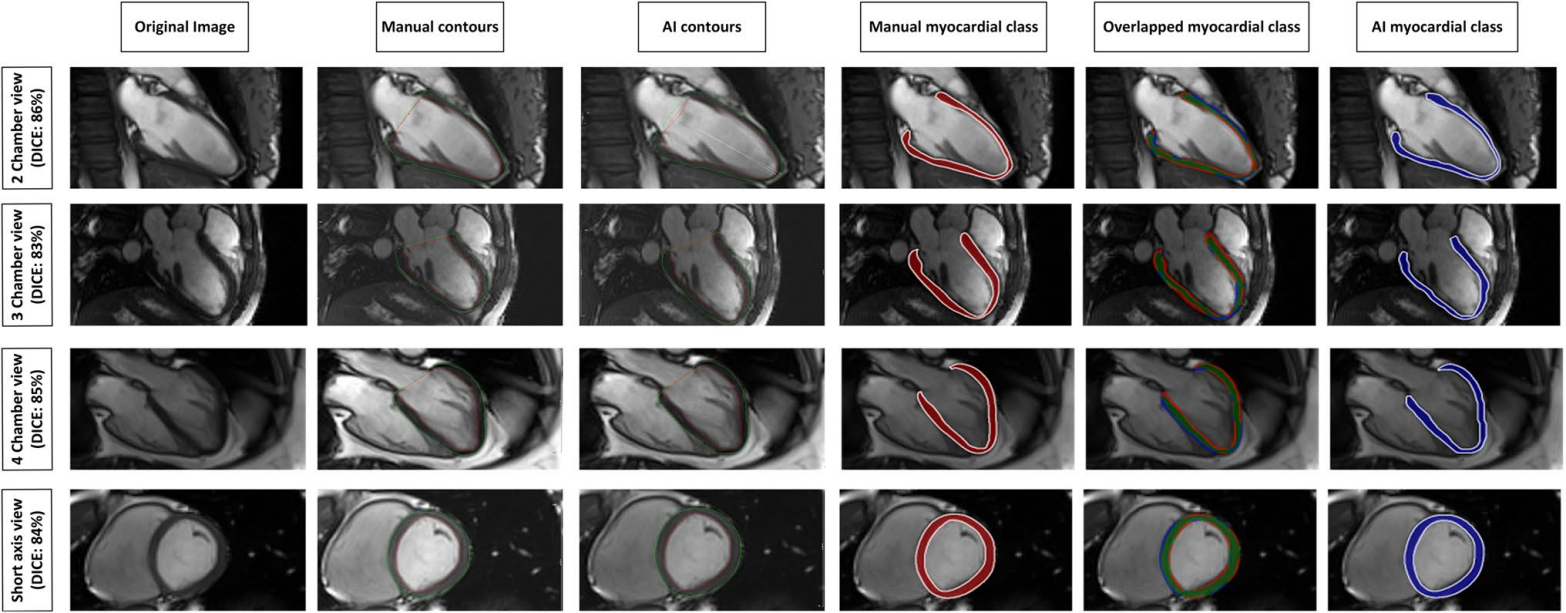
Dice metric comparison between manual and AI segmentations. Comparison of manual (second column from the left) and AI-based segmentations (third column from the left). Third column from the right depicts myocardial class annotations for manual segmentations (red) and the rightest column for AI segmentations (blue). The second column from the right demonstrates the spatial overlap between the contours (green area). Discrepancies are in the corresponding colors of manual (red) or AI contours (blue). DICE values ranged from 83% in the 3-chamber view to 94% in the short axis for the exemplary case

Strain analysis was feasible in all 60 cases for CS, RS, and LS. Global values were as follows: manual (mean % (± SD)) − 16.2 (± 2.2) GCS; 25.5 (± 4.9) GRS; − 17.5 (± 1.8) GLS; and for the AI approach: − 16.7 (± 2.2) GCS; 26.6 (± 5.0) GRS; − 17.3 (± 1.7) GLS. Segmental strain values with standard deviations are presented in Fig. 2. The DSC (in %) showed good agreement between the manual and AI-derived contours with 85.3 ± 10.3 for SAX contours, 85.8 ± 2.9 for 2cv contours, 83.1 ± 5.1 for 3cv contours, and 84.1 ± 4.1 for 4cv contours (Fig. 3). Comparison of the insertion points revealed a mean angle difference of 5.1 ± 10.9° (in relative values 1.4 ± 3.0% difference). Regarding both approaches, the highest CS and LS values as well as the lowest RS values were confined to segments 11 or 12 (insertion point of the papillary muscle) of the AHA model.

### Strain analysis—patients with various cardiac diseases

**Fig. 4:**
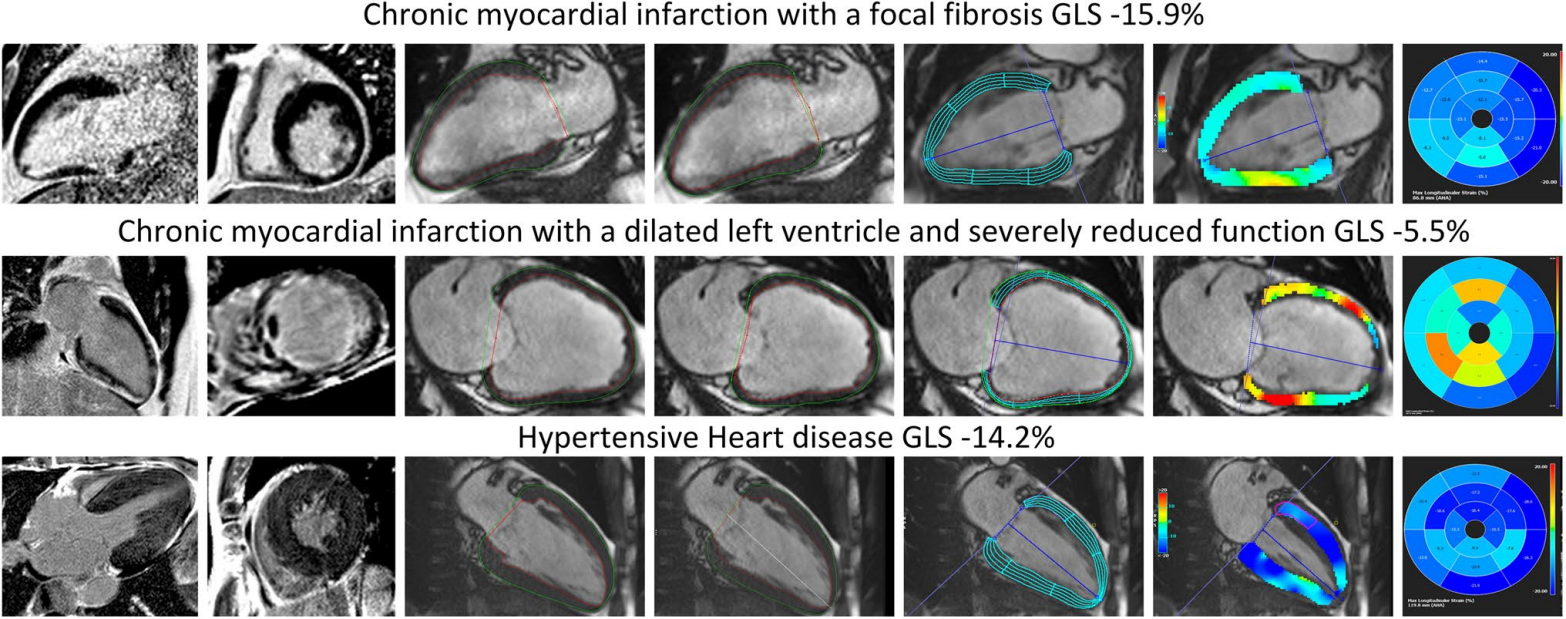
Examples of proper AI contours with strain values and underlying pathologies. Top row represents a patient with a chronic myocardial infarction and focal subendocardial scar at the inferolateral wall (late gadolinium enhancement (LGE)) images in a 2 chamber views (top row, left image) and short axis (top row, second from the left) with subtle wall motion abnormalities. Global longitudinal strain was reduced (− 15.9%). Middle row represents a patient with chronic myocardial infarction and microvascular obstruction on LGE imaging (middle row left and second from the left). Left ventricular function and global longitudinal strains were severely impaired. Bottom row represents a patient with left ventricular hypertrophy due to long-standing arterial hypertension. On LGE imaging a diffuse fibrotic process is visible (bottom row left and second from the left). Global longitudinal strain is mildly reduced with no focal accentuation. The second and third columns from the left represent manual and AI segmentations of two chamber views, respectively

**Fig. 5:**
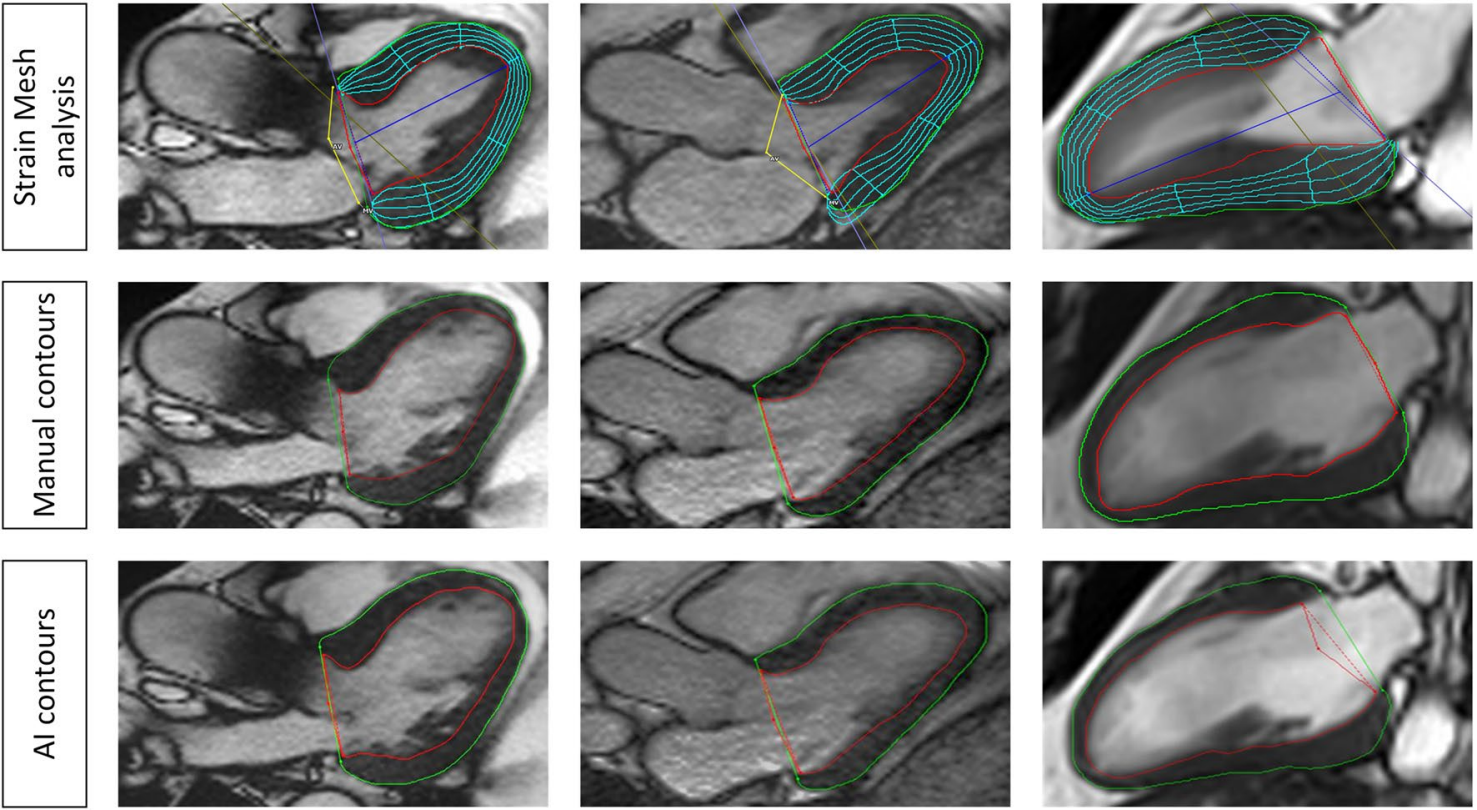
Examples of tracking errors. The left and the middle column show tracking errors in the 3-chamber view at the insertion point of the papillary muscle in the anterolateral segment. Middle and bottom row represent manual and AI segmentations respectively. The right column shows a tracking error in the 2-chamber view in the inferior midventricular segment. Middle and bottom row represent manual and AI segmentations, respectively

AI-derived contour generation and strain analysis was possible in all clinical cases. Global and segmental values are presented in Supplementary Material 1. The Dice metric values showed good agreement between the manual and AI-derived contours with 80.8 ± 9.6 for SAX contours, 85.1 ± 4.6 for 2cv contours, 85.9 ± 6.7 for 3cv contours, and 85.5 ± 4.4 for 4cv contours on average (Table 2). Angular differences for the insertion points were on average 4.4 ± 9.4 degrees (1.2 ± 2.6%) for the cohort with cardiac disease. For the individual pathologies, the results were the following: AHT 3.3 ± 6.4° (0.9 ± 1.8%), AS 2.0 ± 6.5° (0.7 ± 1.8%), HCM 0.3 ± 12.7° (0.01 ± 3.5%), CMI-F 8.3 ± 7.7° (2.3 ± 2.1%), CMI-WF 5.9 ± 8.6° (1.6 ± 2.4%), and CMI-EWF 9.6 ± 9.2° (2.7 ± 2.6%). Figure 4 presents examples with proper tracking and corresponding pathologic features. Tracking errors were found in 27 cases (35.5%; 27/76) of which 3 were from CMI-WF and 24 from the LVH group with 4 in the AHT group (16.7%; 4/24), 16 in the AS group 66.7%, 16/24) and 4 in the HCM group (16.7%; 4/24). Further analysis revealed that tracking errors in the LVH group were mostly confined to the basal, midventricular anterolateral, and inferolateral segments at the insertion point of the papillary muscle (81.5%; 22/24), leading to higher LS strain values in these segments. The inferior midventricular segment was the third most affected region (8.3%; 2/24). In one case from the CMI group, despite proper epicardial contours, tracking features were identified within the right ventricle (3.7%; 1/76). Figure 5 shows examples of tracking errors encountered in the clinical cohort.

**Table 2:** Spatial overlap metrics for the pathologies sorted by short and long axes

**Parameter**		**Chronic myocardial infarction (*N* = 30)**			**Left ventricular hypertrophy (*N* = 46)**		
Subgroup	Healthy volunteers	Focal fibrosis, no WMA and preserved LVEF	Focal fibrosis, WMA and reduced LVEF	Focal fibrosis, global WMA and dilated LV with reduced LVEF	Arterial hypertension	Aortic stenosis	Hypertrophic cardiomyopathy
Dice SAX (%)	85.7 ± 8.6	79.7 ± 9.7	78.4 ± 9.8	78.6 ± 10.7	83.9 ± 8.5	81.5 ± 8.2	81.2 ± 10.5
HD SAX (mm)	2.6 ± 1.2	3.6 ± 1.3	4.0 ± 1.4	4.5 ± 2.1	3.9 ± 1.2	4.0 ± 1.5	4.1 ± 1.3
Dice 2 chamber view (%)	85.8 ± 2.9	83.6 ± 4.5	83.3 ± 3.0	80.5 ± 5.8	87.5 ± 1.9	87.1 ± 3.8	86.6 ± 3.9
HD 2 chamber view (mm)	4.7 ± 2.7	5.2 ± 2.9	6.7 ± 2.2	7.7 ± 4.7	4.4 ± 1.2	4.5 ± 1.7	5.1 ± 2.6
Dice 3 chamber view (%)	83.1 ± 5.1	84.4 ± 2.7	85.4 ± 4.0	77.7 ± 9.3	86.9 ± 2.9	86.4 ± 4.3	85.8 ± 4.3
HD 3 chamber view(mm)	6.7 ± 4.7	5.5 ± 1.9	3.7 ± 1.0	6.4 ± 2.9	4.7 ± 1.7	3.9 ± 1.7	5.1 ± 1.8
Dice 4 chamber view (%)	84.1 ± 4.1	83.6 ± 1.7	83.5 ± 4.4	82.5 ± 4.9	86.2 ± 2.9	87.6 ± 3.4	84.8 ± 3.5
HD 4 chamber view (mm)	7.0 ± 0.9	9.1 ± 4.8	10.0 ± 3.3	8.9 ± 3.3	6.3 ± 2.5	4.8 ± 4.5	6.1 ± 3.8

*HD* Hausdorff distance, *WMA* wall motion abnormalities, *LVEF* left ventricular ejection fraction, *SAX* short axis

### Analysis of images pre- and post-contrast media application

GRS and GCS were lower post-CM for manual segmentations (GRS pre-CM 25.7 ± 6.9% vs. 20.1 ± 7.8 post-CM application; *p* < 0.001; GCS pre-CM − 15.0 ± 3.0 vs. − 13.3 ± 4.2 post-CM; *p* < 0.001) and AI segmentations (GRS pre-CM 28.2 ± 6.8% vs. 21.4 ± 8.1 post-CM; *p* < 0.001; GCS pre-CM − 17.1 ± 2.8 vs. − 13.0 ± 4.3 post-CM; *p* < 0.001).

The DSC and HD metrics were in the same range for pre-CM and post-CM SAX segmentations; however, a lower score post-CM were noted (DSC: 83.6 ± 9.3% for pre-CM vs. 80.5 ± 14.3% post-CM; HD: 3.3 ± 1.4 mm pre-CM vs. 3.6 ± 1.7 mm post-CM).

## Discussion

The main results of our studies are as follows: strain analysis by FT on cine images based on AI-derived contours is feasible and results in equivalent global and segmental strain values with the exception of lateral segments in hypertrophied ventricles. The difference however is attributable to tracking errors as the spatial overlap metric shows good agreement of the methods.

CMR has become the gold standard for LV and right ventricular volume and mass quantification [28] with a standardized approach for analysis and post-processing of images [29]. Yet, there are no consensus recommendations on how to quantify LV myocardial tissue dynamics and deformation by applying CMR. Therefore, we explored how strain assessment by FT can potentially become more standardized involving AI-powered approaches. In our study, we could demonstrate that AI-generated contours for strain assessment by FT are reliable and result in equivalent global and segmental values. In a previous study by Ruijsink et al, DSC between manual and AI-based segmentation was 93% for the endocardial segmentation and 84% for the epicardial segmentation [18]. We found a similar DSC for the myocardial class in our study. Other segmentation algorithms which were tested on the “Automatic Cardiac Diagnosis Challenge” (ACDC) dataset, achieved DSC scores up to 96% for the LV [30]. These scores are higher than the one presented here; however, the underlying dataset in the challenge plays an important factor. The ACDC dataset included similar pathologies as in this study, such as HCM, CMI, and dilated cardiomyopathies; however, whether scans were carried out after contrast administration was not clearly depicted. Images obtained after contrast media application pose an additional challenge as myocardial fibrosis might be mistaken for the blood pool by AI algorithms as well as human readers. In this study, we found the overall lowest segmentation overlap, indicated by DSC and HD metrics, in the CMI group. As all scans in the CMI group were carried out after contrast administration and each case included at least one focal fibrosis; these scans posed a challenge for the algorithm. This was also evidenced by the lower DSC and HD in the comparison of pre-CM and post-CM images. In the subset of LVH, we found higher DSC scores in comparison to the healthy and CMI cohorts. We believe that this paradoxon can be explained by the larger LVM in the LVH cohort as the overall differences in segmentations are divided by a larger area. When considering the HD metric, the healthy cohort showed the lowest value regarding the SAX segmentations.

When segmental values, which are defined according to the AHA model, are compared, the insertion point has to be taken into consideration. In order to verify the proper insertion, visual analysis can be carried out; however, for large data, this is tedious. We propose therefore a comparison based on angular differences as outlined in the methods. The highest angular differences (8.7°) were seen in the CMI-F cohort. Comparing this to the general division of the AHA segments (60°), we feel that these are neglectable; however, the relevance of angular differences and their impact on AHA segments ought to be investigated further.

Interestingly, the study by Ruijsink et al used 3 SAX slices as well as 2cv and 4cv for strain assessment with diastolic contours. This might potentially impact the strain analysis of CS and RS values [15]. Previous studies reported normal values for FT on the full SAX coverage [5, 31]. To additionally achieve a more streamlined post-processing of CMR images, AI algorithms, with the placement of contours in ED and ES for functional assessment, can “recycle” these contours for strain assessment by FT. Further studies employing this approach, potentially in clinical routine scenarios, are needed to verify whether this approach is feasible in a real-world setting. We analyzed not only a healthy population but also one with different clinical entities, which were chosen as either to be a challenge regarding the segmentation (post-contrast cines with fibrosis) or for the FT algorithm (wall motion abnormalities) or both (LVH subgroup, CMI-EWF). In addition, we compared all cases 1:1 and not only selected cases, which minimizes the possibility of errors. The LVH group, especially AS and HCM cases, was burdensome for the AI on a contour and tracking level. Studies about AS and CMR FT have been previously published but none covered AI-derived contours or analysis of tracking errors [32–37]. As the majority of tracking issues in the LVH group was related to the anterolateral and inferolateral segments, papillary muscle hypertrophy could have played a role [38, 39]. As the FT algorithm relies on recognition of voxel-based features, which are in general sparse or even absent in the normal myocardium [40], hypertrophy of the myocardial tissue can further deteriorate tracking. We found the highest CS and LS as well as the lowest RS value in the abovementioned segments in the healthy cohort. Andre et al presented similar results, with the highest variance in these segments. Potentially, the movement of the papillary muscle can have an impact on the values in these segments even in non-pathologic states, which would be further exacerbated with hypertrophy of the myocardium and its appendages. A previous study found statistically significant differences between healthy male and female volunteers in AHA segment 5 of LS analysis [15]. One other study reported a significantly larger papillary muscle mass in males compared to females [41], which might possibly explain the previous findings and the ones presented here. In concordance with the identified segments that pose a challenge for the FT algorithm, the AI-derived strain values show the only significant differences compared to manual contours in these segments. Interestingly, the differences between the methods were due to strain values for AS and HCM cases (Supplementary Material 2). These differences however are not based on a contour level as evidenced by the DSC. Potential influencing factors might relate to the LAX extent or the acquisition itself. In comparison to the other pathologies, we found the 3cv slice location in this group frequently at a narrower angle. This finding might be related to a prominent and dilated ascending aortic root in AS patients impairing proper 3cv acquisitions [42, 43].

The intersegmental differences are a drawback for FT-derived strain values. In a head-to-head comparison between fast-SENC, tagging, and FT, all techniques had good reproducibility; however, in a segmental inter-study comparison, FT showed the lowest agreement [3]. This was confirmed by other studies reporting a rather large variation across segments rendering comparisons rather unfeasible and additionally demonstrating that segmental analysis with FT is complex and clinical implications uncertain [31, 44]. A potential solution might be the use of regional instead of segmental values [45]. In contrast to FT, other techniques such as DENSE have a higher reproducibility of segmental strain values [46, 47]. In addition, segmental strain values provided by DENSE have been shown to carry a prognostic implication in patients after an acute myocardial infarction [48]. Regarding the segmental approach, SENC-derived strain values similarly show a better intersegmental agreement [3, 49]. The fast SENC technique seems highly reproducible, even across different sites [50, 51]. Clinical application of this technique has shown clinical merit; however, more research is needed regarding segmental values [52]. In general, all strain values derived from commercially available software are potentially limited in their comparability as new versions provide new values; hence, providing the software version applied is of great importance.

Lastly, we want to comment on the effect of contrast media application on FT-derived strain values. On the one hand, we noticed that the segmentation becomes more challenging for the AI algorithm; on the other hand, post-CM strain values are lower. This is in line with previous literature [53]. As SAX acquisition are now most of the time acquired after contrast media application, challenges and AI segmentation networks have to take this into consideration.

### Limitations

This is a single-center study with a limited number of cases but reflects different disease entities. This limits potential statistical power in the detection of significant differences in the pairwise comparisons. Another limitation is that we did not compare cardiac contours along the cardiac cycle in order to depict the proper tracking of the FT algorithm. Furthermore, we want to point out that we did not use another vendor, which reduces generalizability.

## Conclusions

Our study shows that application of AI-derived contours for feature tracking and strain analysis in CMR yields results comparable to manual segmentations. Attention should be taken while evaluating left ventricle hypertrophy cases especially in patients with aortic stenosis independent of the use of manual or AI-derived contours.

### Supplementary Information

Below is the link to the electronic supplementary material.

Supplementary file1 (PDF 170 KB)

## Abbreviations

ACDC: Automatic Cardiac Diagnosis Challenge
AHA: American Heart Association
AHT: Arterial hypertension
AI: Artificial intelligence
AS: Aortic stenosis
CMI: Chronic myocardial infarction
CMI-EWF: CMI with reduced LVEF, dilated LV, and global WMA
CMI-F: CMI with focal fibrosis
CMI-WF: CMI with focal fibrosis, reduced LVEF, and regional WMA
CMR: Cardiovascular magnetic resonance
CNN: Convolutional neural network
CS: Circumferential strain
cv: Chamber view
DENSE: Displacement encoding with stimulated echoes
DSC: Dice similarity coefficient
ED: End-diastole
FT: Feature tracking
GCS: Global circumferential strain
GLS: Global longitudinal strain
GRS: Global radial strain
HCM: Hypertrophic cardiomyopathy
HD: Hausdorff distance
LAX: Long axis
LL: “Lazy Luna” tool
LS: Longitudinal strain
LV: Left ventricle
LVEF: Left ventricular ejection fraction
LVH: Left ventricular hypertrophy
LVOT: Left ventricular outflow tract
post-CM: Post-contrast media application
pre-CM: Pre-contrast media application
RS: Radial strain
SAX: Short axis
SD: Standard deviation
SENC: Strain Encoding Magnetic Resonance imaging
WMA: Wall motion abnormalities

## Declarations

### Guarantor

The scientific guarantor of this publication is Edyta Blaszczyk.

### Statistics and biometry

Carsten Schwenke kindly provided statistical advice for this manuscript.

### Study subjects or cohorts overlap

Some study subjects or cohorts have been previously reported in:

• Lim C, Blaszczyk E, Riazy L, et al (2020) Quantification of myocardial strain assessed by cardiovascular magnetic resonance feature tracking in healthy subjects-influence of segmentation and analysis software. Eur Radiol. https://doi.org/10.1007/s00330-020-07539-5

• von Knobelsdorff-Brenkenhoff F, Hennig P, Menza M, et al (2016) Myocardial dysfunction in patients with aortic stenosis and hypertensive heart disease assessed by MR tissue phase mapping. J Magn Reson Imaging 44:168–177. https://doi.org/10.1002/jmri.25125

• Prothmann M, von Knobelsdorff-Brenkenhoff F, Töpper A, et al (2016) High Spatial Resolution Cardiovascular Magnetic Resonance at 7.0 Tesla in Patients with Hypertrophic Cardiomyopathy—First Experiences: Lesson Learned from 7.0 Tesla. PLoS One 11:e0148066. https://doi.org/10.1371/journal.pone.0148066

• Gröschel J, Ammann C, Zange L, et al (2022) Fast acquisition of left and right ventricular function parameters applying cardiovascular magnetic resonance in clinical routine—validation of a 2-shot compressed sensing cine sequence. Scand Cardiovasc J 56:266–275. https://doi.org/10.1080/14017431.2022.2099010

### Methodology

• Retrospective.

• cross-sectional study.

• multicenter study.
