# Supplementary material for: Comparison of manual and artificial intelligence based quantification of myocardial strain by feature tracking—a cardiovascular MR study in health and disease
Source: Eur Radiol. 2023 Aug 18;34(2):1003–15. doi: 10.1007/s00330-023-10127-y (PMC10853310; doi:10.1007/s00330-023-10127-y)

Comparison of manual and artificial intelligence based quantification of myocardial strain by feature tracking - A cardiovascular MR study in health and disease

Electronic Supplementary Material

Supplementary material 1: Table with global and segmental values for the healthy volunteers and the pathologies

Parameter			Chronic myocardial infarction (N=30)						Left ventricular Hypertrophy (N=46)						*p* value*
	Healthy Volunteers		Focal fibrosis. no WMA and preserved LVEF		Focal fibrosis. WMA and reduced LVEF		Focal fibrosis. global WMA and dilated LV with reduced LVEF		Arterial hypertension		Aortic stenosis		Hypertrophic cardiomyopathy		
**Approach**	**Manual**	**AI**	**Manual**	**AI**	**Manual**	**AI**	**Manual**	**AI**	**Manual**	**AI**	**Manual**	**AI**	**Manual**	**AI**	
**GLS**	-17.5 ± 1.8	-17.3 ± 1.7	-15.2 ± 3.1	-15 ± 2.9	-12.2 ± 4.4	-12.6 ± 4.8	-5.4 ± 2	-5 ± 1.9	-15 ± 1.6	-15.7 ± 1.5	-14 ± 3.2	-14.4 ± 3.1	-13.3 ± 4	-14.2 ± 3.9	0.54
**LS AHA 1**	-16.1 ± 5.5	-16.5 ± 6	-14.4 ± 7	-15.3 ± 6	-13.1 ± 5.4	-12.4 ± 5.3	-6.3 ± 2.6	-6.9 ± 2.8	-15.4 ± 4.5	-17.3 ± 6.1	-12.5 ± 7.8	-13.7 ± 4.2	-17.1 ± 5.4	-17.2 ± 4.3	0.46
**LS AHA 2**	-17.8 ± 7.3	-19.2 ± 6.5	-14.8 ± 9.4	-15 ± 4.7	-12.1 ± 5.1	-11.8 ± 8.5	-5.7 ± 5	-5.2 ± 7.9	-9.5 ± 9.1	-10.9 ± 7.8	-5.7 ± 11.2	-9.6 ± 8.7	-10.9 ± 8.3	-10 ± 8.8	0.52
**LS AHA 3**	-17.2 ± 4.8	-17.7 ± 5.8	-13.2 ± 4.9	-12.3 ± 3.3	-11.2 ± 4.5	-10.5 ± 7.6	-7.6 ± 2.6	-5.7 ± 4.8	-12.5 ± 8.3	-14 ± 5.6	-10.9 ± 7.9	-11.2 ± 6.1	-10.7 ± 5	-12 ± 6	0.98
**LS AHA 4**	-24.1 ± 4.9	-24.3 ± 5	-20.2 ± 4.9	-17.6 ± 7.6	-16.6 ± 6.4	-19 ± 5.7	-12.3 ± 5.6	-7.9 ± 7.3	-21.9 ± 4.7	-22.8 ± 3.7	-20.5 ± 6	-18.3 ± 7.5	-15.8 ± 11.9	-18.3 ± 6.8	0.59
**LS AHA 5**	-27.1 ± 4.1	-27.2 ± 6.5	-23.5 ± 7.3	-23.6 ± 4.1	-23.5 ± 5.2	-21.5 ± 6.3	-15.7 ± 4.9	-14.5 ± 5.4	-26.4 ± 2.8	-26.9 ± 3	-23.3 ± 4.3	-22.6 ± 4.3	-24.6 ± 5.6	-25.8 ± 5.3	0.69
**LS AHA 6**	-24.6 ± 5.1	-24.9 ± 7.3	-20 ± 3.9	-19.1 ± 5.5	-19.8 ± 5.3	-20 ± 4	-10.3 ± 7.4	-10.4 ± 5.6	-25.3 ± 3.8	-26.4 ± 2.7	-22.1 ± 4	-23.7 ± 3.9	-24.6 ± 6.2	-23.7 ± 6.6	0.81

**LS AHA 7**	-19.5 ± 5.8	-18.5 ± 7.6	-13.6 ± 4	-13.2 ± 4.5	-9.8 ± 8.7	-12.4 ± 5.3	-5.2 ± 5.7	-6.7 ± 3.1	-13.8 ± 9.9	-15 ± 6.4	-13.8 ± 7.9	-11 ± 10.4	-14.1 ± 10.2	-13.2 ± 10.5	0.99
**LS AHA 8**	-18.1 ± 4.3	-16.8 ± 5.1	-17.7 ± 4.1	-16.9 ± 4.6	-14.3 ± 6.6	-14.8 ± 6.9	-1.8 ± 4.5	-2.6 ± 5.6	-17.3 ± 2.2	-18.7 ± 2.1	-17 ± 4.6	-18.2 ± 4.9	-12.8 ± 8.9	-13.2 ± 9.3	0.71
**LS AHA 9**	-15.6 ± 7	-13.5 ± 7.2	-14.7 ± 5.4	-14.6 ± 4.7	-10.7 ± 6.3	-9.3 ± 8	1 ± 7	-3.3 ± 4.4	-10.2 ± 7.1	-11.8 ± 6.9	-11.8 ± 5.7	-12.4 ± 7.1	-9.5 ± 8.8	-10.9 ± 7.3	0.53
**LS AHA 10**	-14.4 ± 5.4	-11.8 ± 7.4	-11.8 ± 10.2	-8.8 ± 12.1	-8.4 ± 7.2	-6.4 ± 7.4	-4.2 ± 4	-2.2 ± 3.6	-6.9 ± 11.1	-6.2 ± 8.9	-6.3 ± 8	-5.9 ± 8.8	-3.7 ± 13.8	-0.2 ± 15.1	0.11
**LS AHA 11**	-8.8 ± 12.2	-10.3 ± 12.8	-10.2 ± 9.8	-10.4 ± 11.9	-1.3 ± 10.5	-3.9 ± 13.1	-2.2 ± 6.7	-3.5 ± 5.2	-4.3 ± 14.2	-4.4 ± 12.7	-6.2 ± 9.6	-5.8 ± 9.9	-8.1 ± 13.9	-8.6 ± 11.9	0.63
**LS AHA 12**	-15.6 ± 7.7	-15.9 ± 9.9	-16.3 ± 8.9	-16.7 ± 8.1	-13 ± 6.9	-7.8 ± 13.8	-3.8 ± 5.2	-2.7 ± 3.9	-9.1 ± 18	-6.1 ± 17.7	-11 ± 14	-14.5 ± 8.8	-7.9 ± 20.5	-7.8 ± 17.7	0.71
**LS AHA 13**	-17.9 ± 6.2	-17 ± 4.9	-16.3 ± 4.1	-16.2 ± 3.6	-11.1 ± 6.9	-13.2 ± 6.3	-5.4 ± 5.1	-3.8 ± 5.4	-15.1 ± 4.9	-15.2 ± 3.8	-14.4 ± 4.8	-15.3 ± 5.3	-13.7 ± 8.7	-14.8 ± 8.4	0.79
**LS AHA 14**	-15.7 ± 5	-14.2 ± 8.3	-13.8 ± 8.4	-16.4 ± 4	-12.3 ± 7.9	-12.2 ± 7.4	-4.2 ± 7.1	-4.2 ± 5.2	-17.9 ± 4.4	-18.2 ± 5	-16.2 ± 4.3	-16.8 ± 3.6	-16.6 ± 9.5	-16.4 ± 9.2	0.82
**LS AHA 15**	-15.7 ± 4.3	-12.9 ± 5.4	-13.8 ± 3.5	-13.4 ± 2.6	-9.8 ± 6.5	-10.4 ± 8.2	-0.3 ± 5.4	-3.8 ± 3.8	-8.5 ± 8	-6.9 ± 9.3	-11.3 ± 3.6	-11.1 ± 4.6	-9.2 ± 12.4	-10.5 ± 11.2	0.94
**LS AHA 16**	-13.8 ± 6.3	-14.3 ± 5.9	-11.1 ± 5.6	-11.1 ± 5.1	-7.7 ± 9.1	-8.5 ± 10.8	-1.7 ± 6.3	-0.5 ± 7.3	-12.5 ± 7.8	-10.4 ± 8.7	-10.3 ± 7.1	-10.7 ± 4	-10.3 ± 10.4	-11.2 ± 11.7	0.93
**GRS**	25.5 ± 4.9	26.6 ± 5	22.2 ± 5	23 ± 5.3	15.3 ± 6.1	16.6 ± 6.2	6.4 ± 2.1	6.5 ± 2.2	19.9 ± 5.8	23.4 ± 6.4	22.5 ± 8.3	25.5 ± 8.2	25.8 ± 7.8	29.3 ± 7.8	**0.03**
**RS AHA 1**	30.3 ± 9.3	31.6 ± 9.5	23.3 ± 8.2	27.6 ± 10.4	20.8 ± 6.9	21 ± 7.7	10.2 ± 2	13 ± 5.7	24.4 ± 7.9	23.8 ± 8.6	30.2 ± 11.2	30.7 ± 10.5	34.7 ± 14.2	34.1 ± 16.4	0.47

**RS AHA 2**	27.7 ± 8.1	29 ± 7.8	21.8 ± 8.1	23.6 ± 7.4	14.8 ± 9.6	17.7 ± 10.8	6.2 ± 2.8	5.5 ± 1.9	19.4 ± 7.6	21 ± 7.6	21.8 ± 10.1	22.9 ± 11.8	22.6 ± 9.8	21.5 ± 10.1	0.44
**RS AHA 3**	27.8 ± 8.2	29.5 ± 9.1	19.5 ± 6.8	19.3 ± 6.2	10.9 ± 11.2	11.3 ± 10.7	5.4 ± 5.3	7.4 ± 3.8	17 ± 5.1	17.9 ± 5.3	18.5 ± 9	19.6 ± 9.2	21.6 ± 9	21.5 ± 10.6	0.51
**RS AHA 4**	22.6 ± 7.2	24.1 ± 8	19.1 ± 6.2	17.9 ± 5.1	10.8 ± 9.3	10.3 ± 8.9	8.8 ± 7.4	7.2 ± 8	16.6 ± 6.8	18.8 ± 5	22.2 ± 10.1	23.9 ± 9.3	24.8 ± 8.2	27.8 ± 7.8	0.54
**RS AHA 5**	27 ± 10.7	27.3 ± 12.8	21.7 ± 8	24.2 ± 8.1	15.8 ± 5.4	16 ± 5.9	13.8 ± 6.7	13.8 ± 7.7	22 ± 13.6	25.5 ± 12.1	25.9 ± 11	34 ± 12	29.1 ± 12.5	38 ± 12.5	**0.045**
**RS AHA 6**	30.2 ± 10.3	28.6 ± 13.9	22.3 ± 11.8	28.2 ± 14.9	18.6 ± 10.3	20.7 ± 7.8	14.2 ± 9	14.1 ± 8.7	23.8 ± 11.7	26.6 ± 10.9	26.1 ± 10.3	30.4 ± 10.6	35.7 ± 14.5	36.4 ± 12.3	0.25
**RS AHA 7**	26.8 ± 9.3	26.5 ± 11.3	24.4 ± 7.3	24 ± 6.7	18.2 ± 11.1	18.6 ± 8.9	8.4 ± 4	9.4 ± 5.6	22.7 ± 8.9	27.6 ± 9.3	24.7 ± 11.7	26.8 ± 10.4	26.4 ± 13.2	29.3 ± 10.3	0.31
**RS AHA 8**	27.5 ± 8	27.1 ± 12.6	28 ± 7.8	28.1 ± 7.4	19.8 ± 12.9	22 ± 9.4	5 ± 5.2	5.9 ± 4.1	27 ± 7.3	29.2 ± 8.2	31.4 ± 11.6	31.8 ± 9.8	30.9 ± 10.7	34.9 ± 10.5	0.37
**RS AHA 9**	30.6 ± 7.3	29 ± 12.9	25.7 ± 5.8	26.5 ± 4.7	17.4 ± 7.3	19.2 ± 6.9	5.3 ± 4.1	5.6 ± 4.2	22.7 ± 8.6	27 ± 6.8	26.1 ± 11.6	28.6 ± 11	37.8 ± 19.1	41.9 ± 17.8	0.29
**RS AHA 10**	22.4 ± 6.3	21 ± 8.8	21 ± 5.7	21 ± 6	13.1 ± 6.1	13.6 ± 6.4	8.9 ± 6	7 ± 3.6	12.8 ± 3.6	20 ± 8.9	14.1 ± 7.3	20.1 ± 8.4	19.1 ± 11	25.8 ± 11.3	**0.03**
**RS AHA 11**	19.1 ± 5.9	18.2 ± 8.7	17.3 ± 6	18.8 ± 7	10.8 ± 3.1	13.9 ± 7.6	8.1 ± 3.9	9.1 ± 4.9	13.3 ± 8.5	19.8 ± 10	13.2 ± 5.6	18.2 ± 7.2	16.2 ± 9.8	23.3 ± 7.3	**0.002**
**RS AHA 12**	17.5 ± 6.3	17.8 ± 9.1	16 ± 7.7	19 ± 9.7	10.2 ± 7	13.5 ± 9.3	7.4 ± 4.2	7.9 ± 5.4	14 ± 8.3	18.8 ± 9.8	15.4 ± 6.9	18.8 ± 9.1	16.8 ± 7.4	19.8 ± 7.5	**0.03**
**RS AHA 13**	24.5 ± 9.9	27.3 ± 13.3	34.4 ± 7.4	26.3 ± 7.7	19.5 ± 12.1	19.1 ± 10.5	3.9 ± 9.9	3.1 ± 5.8	30.5 ± 11	27.1 ± 10.4	27.9 ± 10.8	29.2 ± 9.1	29.8 ± 19.1	31.5 ± 14.2	0.57

**RS AHA 14**	32.2 ± 9.1	38.3 ± 11.9	35.5 ± 12.2	31.4 ± 8.4	22.8 ± 10.9	22 ± 10.5	6.4 ± 10.4	3.9 ± 7	34.4 ± 10	30.5 ± 11.8	33.2 ± 10.8	34.4 ± 9.3	46 ± 20.1	45.1 ± 15.7	0.68
**RS AHA 15**	41 ± 11.7	40.2 ± 17.3	34.9 ± 10.7	31.9 ± 10.9	21.4 ± 11.4	22 ± 10	4 ± 6.2	3.6 ± 7.2	25 ± 7.4	32.3 ± 11.2	25.7 ± 16.4	33.4 ± 11.4	39.8 ± 24.5	45.8 ± 22.5	0.27
**RS AHA 16**	27.4 ± 12	25.6 ± 16.3	29.4 ± 12.6	26.8 ± 12.9	15.3 ± 8.9	17.8 ± 10.5	4 ± 6.1	2.4 ± 5.9	25.6 ± 14.4	28.7 ± 13.5	19.8 ± 11.1	28.1 ± 10.8	22.3 ± 15.2	33.2 ± 17.3	0.18
**GCS**	-16.2 ± 2.2	-16.7 ± 2.2	-14.8 ± 2.5	-15.2 ± 2.7	-11 ± 3.5	-11.8 ± 3.4	-5.3 ± 1.5	-5.4 ± 1.5	-13.5 ± 2.9	-15.2 ± 2.9	-14.6 ± 3.9	-16 ± 3.6	-15.5 ± 3.2	-17 ± 3.2	**0.02**
**CS AHA 1**	-18 ± 3.6	-18.6 ± 3.4	-15.2 ± 4.1	-16.9 ± 4	-14 ± 3.4	-14.1 ± 4	-8.1 ± 1.2	-9.8 ± 3.4	-15.7 ± 3.8	-15.4 ± 4.2	-17.9 ± 4.5	-18.1 ± 4.3	-19.2 ± 4.5	-18.5 ± 5.3	0.43
**CS AHA 2**	-17.1 ± 3.5	-17.8 ± 3.2	-14.5 ± 3.9	-15.5 ± 3.3	-10.2 ± 7.1	-11.8 ± 7.4	-5.3 ± 2.1	-4.8 ± 1.5	-13.3 ± 3.7	-14.2 ± 3.4	-14.1 ± 4.8	-14.4 ± 5.5	-14.1 ± 4.6	-13.6 ± 4.6	0.46
**CS AHA 3**	-17.1 ± 3.4	-17.7 ± 3.2	-13.4 ± 3.6	-13.4 ± 3.2	-7.2 ± 8.7	-6.4 ± 9.7	-3.9 ± 5.1	-5.6 ± 3.9	-12.3 ± 2.9	-12.7 ± 2.9	-12.6 ± 4.7	-13.1 ± 4.7	-13.7 ± 3.9	-13.5 ± 4.7	0.62
**CS AHA 4**	-14.8 ± 3.3	-15.4 ± 3.5	-13.3 ± 3.7	-12.8 ± 3	-6.8 ± 7.8	-6.3 ± 8	-6.7 ± 5	-5.4 ± 5.6	-11.8 ± 3.5	-13.2 ± 2.7	-14.4 ± 4.7	-15.4 ± 4.1	-15.6 ± 3.6	-16.8 ± 3	0.66
**CS AHA 5**	-16.4 ± 4.5	-15.4 ± 11.4	-14.5 ± 3.4	-15.7 ± 3.4	-11.5 ± 2.9	-11.6 ± 3.1	-10 ± 4.1	-8.9 ± 6.8	-13.8 ± 5.4	-15.9 ± 4.8	-15.9 ± 4.7	-19.2 ± 4.3	-16.6 ± 4.6	-20.1 ± 4.2	0.26
**CS AHA 6**	-17.8 ± 4.1	-16.2 ± 10.2	-14.2 ± 5.7	-16.6 ± 5.8	-12.5 ± 5.3	-13.9 ± 4	-10.2 ± 5.1	-10.2 ± 5.1	-14.9 ± 5	-16.3 ± 4.1	-16.2 ± 4.7	-17.9 ± 4.2	-18.7 ± 4.4	-19.5 ± 4	0.36
**CS AHA 7**	-16.7 ± 3.8	-15.8 ± 8.4	-15.9 ± 3.5	-15.8 ± 3.1	-12.3 ± 5.5	-12.8 ± 4.4	-6.7 ± 2.7	-7.4 ± 3.6	-15 ± 4.2	-17 ± 4	-15.5 ± 5.3	-16.6 ± 4.6	-15.7 ± 5.1	-17.4 ± 4.3	0.38
**CS AHA 8**	-17.1 ± 3.3	-16.4 ± 7.5	-17.5 ± 3.3	-17.6 ± 3.1	-12.5 ± 8.8	-13.2 ± 8.1	-4 ± 4.8	-4.9 ± 3.6	-17.1 ± 3.1	-17.9 ± 3.4	-18.4 ± 4.7	-18.7 ± 3.9	-17.9 ± 4	-19.1 ± 3.8	0.54

**CS AHA 9**	-18.5 ± 2.8	-17.2 ± 8	-16.6 ± 2.6	-17.1 ± 2	-12.3 ± 3.8	-13.4 ± 3.6	-4.1 ± 3.6	-4.7 ± 3	-14.9 ± 4.6	-17 ± 3.1	-16.2 ± 5	-17.3 ± 4.4	-19.1 ± 5.6	-20.8 ± 5.5	0.28
**CS AHA 10**	-14.9 ± 3	-13.5 ± 6.9	-14.4 ± 2.9	-14.3 ± 3.1	-9.8 ± 3.6	-10.1 ± 3.5	-7 ± 3.9	-5.9 ± 2.5	-9.8 ± 2.2	-13.5 ± 4.3	-10.2 ± 4.4	-13.6 ± 4	-11.7 ± 7.3	-15.5 ± 4.3	0.08
**CS AHA 11**	-13.2 ± 3	-11.6 ± 10.6	-12.4 ± 3.4	-13.1 ± 3.7	-8.6 ± 2.2	-10.3 ± 3.9	-6.5 ± 2.6	-7.2 ± 3	-9.7 ± 4.8	-13.3 ± 4.3	-9.8 ± 3.4	-12.6 ± 3.4	-10.9 ± 6.4	-15 ± 3.5	0.058
**CS AHA 12**	-12.4 ± 3.4	-11.9 ± 8	-11.5 ± 4.3	-13 ± 5	-7.8 ± 5.1	-9.6 ± 5.6	-6 ± 3	-6.4 ± 3.6	-10.2 ± 4.4	-12.8 ± 4.2	-11.2 ± 3.8	-12.8 ± 4.5	-11.7 ± 4.2	-13.2 ± 3.8	0.10
**CS AHA 13**	-15.3 ± 4.7	-15.2 ± 11.7	-19.9 ± 2.5	-16.5 ± 3.4	-12.6 ± 7.7	-12.5 ± 7.2	-0.9 ± 8.2	-2.1 ± 5.4	-18.1 ± 4.5	-16.6 ± 4.1	-16.9 ± 4.4	-17.5 ± 3.6	-15.8 ± 8.9	-17.4 ± 6.2	0.83
**CS AHA 14**	-18.6 ± 3.5	-19.8 ± 8.5	-19.9 ± 4	-18.2 ± 3.5	-14.8 ± 4.9	-14.3 ± 4.6	-4.3 ± 7.7	-2.7 ± 7.1	-19.8 ± 3.8	-17.9 ± 4.4	-19.2 ± 4.3	-19.6 ± 3.4	-21.9 ± 6.5	-21.6 ± 4.9	0.48
**CS AHA 15**	-21.3 ± 3.6	-20.2 ± 8.3	-19.7 ± 4.3	-18.2 ± 4.7	-14 ± 4.9	-14.4 ± 4.4	-1.4 ± 6.4	-2.3 ± 6.9	-15.9 ± 3.5	-18.5 ± 3.9	-15.1 ± 6.4	-18.8 ± 4.3	-18.8 ± 7.3	-21.2 ± 7.4	0.24
**CS AHA 16**	-16.1 ± 5	-14.8 ± 8	-17.4 ± 5.3	-16.1 ± 5.5	-10.2 ± 6.3	-11.9 ± 6.7	-2.9 ± 5.8	-1.5 ± 6	-15.6 ± 6.4	-17 ± 4.7	-12.9 ± 5.8	-16.9 ± 4.6	-13.2 ± 8.5	-17.5 ± 7.1	0.27

WMA= wall motion abnormalities; LV=left ventricle; LVEF= left ventricular ejection fraction; AI= artificial intelligence; GLS=global longitudinal strain; LS= longitudinal strain: AHA= American heart association segment; GRS= global radial strain; RS= radial strain; GCS= global circumferential strain; CS= circumferential strain *a p-value of <0.05 was considered statistically significant; pairwise comparison in revealed significant differences for AS and HCM for RS AHA segment 5 (p=0.01 and p=0.03, respectively), 10 (p=0.01 and p=0.02, respectively), 11 (p=0.02 and p=0.01, respectively).

Supplementary material 2: Whisker plots for AHA strain segments 10 for radial and circumferential strain values

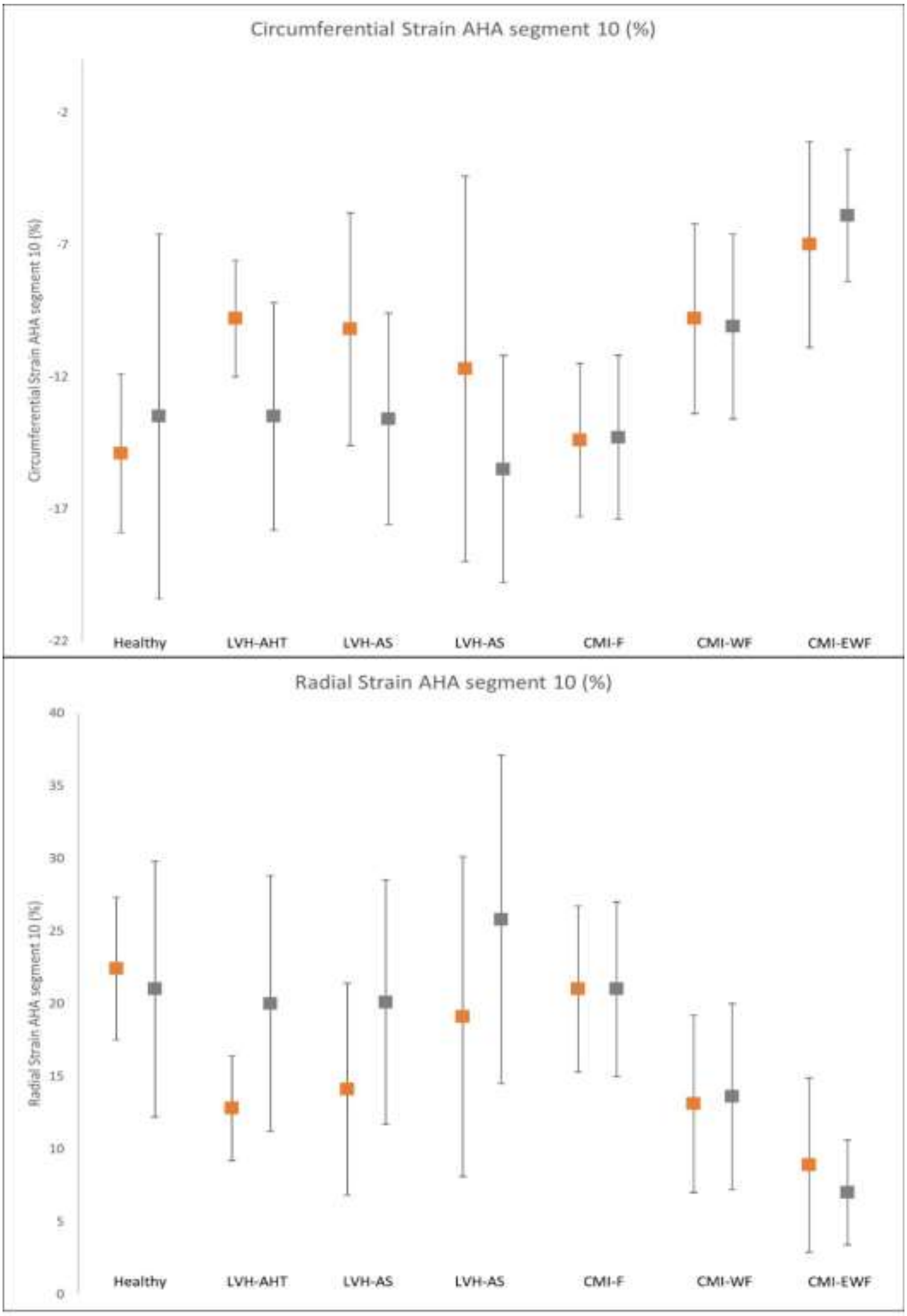

Supplement: Supplementary file 1 — Supplementary file1 (PDF 170 KB) [file 330_2023_10127_MOESM1_ESM.pdf]
